# Supplementary material for: Centromeres in the thermotolerant yeast K. marxianus mediate attachment to a single microtubule
Source: Chromosome Res. 2025 Jul 3;33(1):14. doi: 10.1007/s10577-025-09772-4 (PMC12226651; doi:10.1007/s10577-025-09772-4)
Supplement: Supplementary file 1 — Supplementary file1 (PDF 110 KB) [file 10577_2025_9772_MOESM1_ESM.pdf]

| Accession | Protein description |
|---|---|
| W0TFR3 | Nonsense-mediated decay protein 4 OS=Kluyveromyces marxianus (strain DMKU3-1042 / BCC 29191 / NBRC 104275) OX=1003335 GN=NMD4 PE=4 SV=1 |
| W0TDX5 | 3'(2'),5'-bisphosphate nucleotidase OS=Kluyveromyces marxianus (strain DMKU3-1042 / BCC 29191 / NBRC 104275) OX=1003335 GN=MET22 PE=3 SV=1 |
| W0TH38 | Eukaryotic translation initiation factor 2 subunit alpha OS=Kluyveromyces marxianus (strain DMKU3-1042 / BCC 29191 / NBRC 104275) OX=1003335 GN=SUI2 PE=3 SV=1 |
| Q24FB1 | Squalene-tetrahymanol cyclase THC1 OS=Tetrahymena thermophila (strain SB210) OX=312017 GN=THC1 PE=1 SV=2 |
| W0T6E8 | Sm_like super family OS=Kluyveromyces marxianus (strain DMKU3-1042 / BCC 29191 / NBRC 104275) OX=1003335 GN=LSM7 PE=3 SV=1 |
| W0TFD2 | ATPase inhibitor, mitochondrial OS=Kluyveromyces marxianus (strain DMKU3-1042 / BCC 29191 / NBRC 104275) OX=1003335 GN=KLMA_60492 PE=3 SV=1 |
| W0TI22 | Vacuolar protein sorting-associated protein OS=Kluyveromyces marxianus (strain DMKU3-1042 / BCC 29191 / NBRC 104275) OX=1003335 GN=VPS13 PE=3 SV=1 |
| W0T8I7 | DUF3535 OS=Kluyveromyces marxianus (strain DMKU3-1042 / BCC 29191 / NBRC 104275) OX=1003335 GN=MOT1 PE=4 SV=1 |
| W0T8D8 | V-type proton ATPase proteolipid subunit OS=Kluyveromyces marxianus (strain DMKU3-1042 / BCC 29191 / NBRC 104275) OX=1003335 GN=VMA3 PE=3 SV=1 |
| W0TEM1 | DNA damage-binding protein CMR1 OS=Kluyveromyces marxianus (strain DMKU3-1042 / BCC 29191 / NBRC 104275) OX=1003335 GN=KLMA_70244 PE=3 SV=1 |
| W0T858 | GrpE protein homolog OS=Kluyveromyces marxianus (strain DMKU3-1042 / BCC 29191 / NBRC 104275) OX=1003335 GN=mge1 PE=3 SV=1 |
| W0TGZ2 | ATP-dependent RNA helicase DHH1 OS=Kluyveromyces marxianus (strain DMKU3-1042 / BCC 29191 / NBRC 104275) OX=1003335 GN=DHH1 PE=3 SV=1 |
| W0T4R4 | Chromatin structure-remodeling complex subunit RSC7 OS=Kluyveromyces marxianus (strain DMKU3-1042 / BCC 29191 / NBRC 104275) OX=1003335 GN=NPL6 PE=4 SV=1 |
| W0TA87 | Uncharacterized protein OS=Kluyveromyces marxianus (strain DMKU3-1042 / BCC 29191 / NBRC 104275) OX=1003335 GN=SAE2 PE=4 SV=1 |
| W0TF89 | Chromatin structure-remodeling complex subunit RSC9 OS=Kluyveromyces marxianus (strain DMKU3-1042 / BCC 29191 / NBRC 104275) OX=1003335 GN=RSC9 PE=4 SV=1 |
| W0T4Z8 | Protein RMD9 OS=Kluyveromyces marxianus (strain DMKU3-1042 / BCC 29191 / NBRC 104275) OX=1003335 GN=RMD9 PE=4 SV=1 |
| W0TBR8 | U6 snRNA-associated Sm-like protein LSm3 OS=Kluyveromyces marxianus (strain DMKU3-1042 / BCC 29191 / NBRC 104275) OX=1003335 GN=LSM3 PE=4 SV=1 |
| W0TEY9 | C-1-tetrahydrofolate synthase OS=Kluyveromyces marxianus (strain DMKU3-1042 / BCC 29191 / NBRC 104275) OX=1003335 GN=MIS1 PE=3 SV=1 |

| | |
|---|---|
| W0TGR6 | Conserved hypothetical membrane protein OS=Kluyveromyces marxianus (strain DMKU3-1042 / BCC 29191 / NBRC 104275) OX=1003335 GN=KLMA_70420 PE=4 SV=1 |
| W0TDK4 | Target of rapamycin complex 2 subunit AVO1 OS=Kluyveromyces marxianus (strain DMKU3-1042 / BCC 29191 / NBRC 104275) OX=1003335 GN=AVO1 PE=3 SV=1 |
| W0T7V3 | ISWI chromatin-remodeling complex ATPase ISW1 OS=Kluyveromyces marxianus (strain DMKU3-1042 / BCC 29191 / NBRC 104275) OX=1003335 GN=ISW1 PE=3 SV=1 |
| W0TK27 | Protein MKT1 OS=Kluyveromyces marxianus (strain DMKU3-1042 / BCC 29191 / NBRC 104275) OX=1003335 GN=MKT1 PE=3 SV=1 |
| W0TAP1 | Uncharacterized protein YPR085C OS=Kluyveromyces marxianus (strain DMKU3-1042 / BCC 29191 / NBRC 104275) OX=1003335 GN=ASA1 PE=4 SV=1 |
| W0T5B2 | RSC chromatin remodeling complex subunit RSC8 OS=Kluyveromyces marxianus (strain DMKU3-1042 / BCC 29191 / NBRC 104275) OX=1003335 GN=SWI3 PE=4 SV=1 |
| W0T460 | 37S ribosomal protein NAM9 OS=Kluyveromyces marxianus (strain DMKU3-1042 / BCC 29191 / NBRC 104275) OX=1003335 GN=KLMA_10573 PE=3 SV=1 |
| W0TGJ8 | Ubiquinone biosynthesis protein COQ4, mitochondrial OS=Kluyveromyces marxianus (strain DMKU3-1042 / BCC 29191 / NBRC 104275) OX=1003335 GN=COQ4 PE=3 SV=1 |
| W0THZ6 | U6 snRNA-associated Sm-like protein LSm4 OS=Kluyveromyces marxianus (strain DMKU3-1042 / BCC 29191 / NBRC 104275) OX=1003335 GN=LSM4 PE=3 SV=1 |
| W0TA74 | Putative aldehyde dehydrogenase-like protein YHR039C OS=Kluyveromyces marxianus (strain DMKU3-1042 / BCC 29191 / NBRC 104275) OX=1003335 GN=ALDH22A1 PE=3 SV=1 |
| W0TEU0 | Sorting nexin-41 OS=Kluyveromyces marxianus (strain DMKU3-1042 / BCC 29191 / NBRC 104275) OX=1003335 GN=SNX41 PE=3 SV=1 |
| W0T7P3 | Rtt102p super family OS=Kluyveromyces marxianus (strain DMKU3-1042 / BCC 29191 / NBRC 104275) OX=1003335 GN=KLMA_10502 PE=4 SV=1 |
| W0TA33 | Antiviral helicase SKI2 OS=Kluyveromyces marxianus (strain DMKU3-1042 / BCC 29191 / NBRC 104275) OX=1003335 GN=SKI2 PE=3 SV=1 |
| W0T4T9 | Replication protein A subunit OS=Kluyveromyces marxianus (strain DMKU3-1042 / BCC 29191 / NBRC 104275) OX=1003335 GN=RFA1 PE=3 SV=1 |
| W0TH80 | Chromatin-remodeling ATPase INO80 OS=Kluyveromyces marxianus (strain DMKU3-1042 / BCC 29191 / NBRC 104275) OX=1003335 GN=INO80 PE=3 SV=1 |
| W0TBC9 | Checkpoint serine/threonine-protein kinase BUB1 OS=Kluyveromyces marxianus (strain DMKU3-1042 / BCC 29191 / NBRC 104275) OX=1003335 GN=BUB1 PE=4 SV=1 |
| W0T7F9 | Chromatin structure-remodeling complex protein RSC8 OS=Kluyveromyces marxianus (strain DMKU3-1042 / BCC 29191 / NBRC 104275) OX=1003335 GN=RSC8 PE=4 SV=1 |

| | |
|---|---|
| W0T9X2 | Replication factor A protein 2 OS=Kluyveromyces marxianus (strain DMKU3-1042 / BCC 29191 / NBRC 104275) OX=1003335 GN=RFA2 PE=3 SV=2 |
| W0T916 | Uncharacterized bolA-like protein YAL044W-A OS=Kluyveromyces marxianus (strain DMKU3-1042 / BCC 29191 / NBRC 104275) OX=1003335 GN=KLMA_30590 PE=3 SV=1 |
| W0TDR8 | H2A super family protein OS=Kluyveromyces marxianus (strain DMKU3-1042 / BCC 29191 / NBRC 104275) OX=1003335 GN=BUR6 PE=4 SV=1 |
| W0TJ61 | Heterogeneous nuclear rnp K-like protein 2 OS=Kluyveromyces marxianus (strain DMKU3-1042 / BCC 29191 / NBRC 104275) OX=1003335 GN=HEK2 PE=3 SV=1 |
| W0T941 | Chromatin structure-remodeling complex subunit RSC4 OS=Kluyveromyces marxianus (strain DMKU3-1042 / BCC 29191 / NBRC 104275) OX=1003335 GN=RSC4 PE=4 SV=1 |
| W0TER7 | Nuclear protein STH1/NPS1 OS=Kluyveromyces marxianus (strain DMKU3-1042 / BCC 29191 / NBRC 104275) OX=1003335 GN=STH1 PE=4 SV=1 |
| W0TFQ1 | Mitotic spindle-associated protein SHE1 OS=Kluyveromyces marxianus (strain DMKU3-1042 / BCC 29191 / NBRC 104275) OX=1003335 GN=SHE1 PE=4 SV=1 |
| W0TA47 | DNA-directed RNA polymerase III subunit RPC3 OS=Kluyveromyces marxianus (strain DMKU3-1042 / BCC 29191 / NBRC 104275) OX=1003335 GN=RPC82 PE=3 SV=1 |
| W0T4U9 | Reduced growth phenotype protein 1 OS=Kluyveromyces marxianus (strain DMKU3-1042 / BCC 29191 / NBRC 104275) OX=1003335 GN=RGP1 PE=4 SV=1 |
| W0TG55 | Protein phosphatase 2C homolog 4 OS=Kluyveromyces marxianus (strain DMKU3-1042 / BCC 29191 / NBRC 104275) OX=1003335 GN=PTC4 PE=3 SV=1 |
| W0TKL4 | DNA-directed RNA polymerase subunit OS=Kluyveromyces marxianus (strain DMKU3-1042 / BCC 29191 / NBRC 104275) OX=1003335 GN=RPO31 PE=3 SV=1 |
| W0TFW3 | Actin patch protein 1 OS=Kluyveromyces marxianus (strain DMKU3-1042 / BCC 29191 / NBRC 104275) OX=1003335 GN=APP1 PE=4 SV=1 |
| W0TCE3 | Coatomer subunit epsilon OS=Kluyveromyces marxianus (strain DMKU3-1042 / BCC 29191 / NBRC 104275) OX=1003335 GN=SEC28 PE=3 SV=1 |
| W0TCZ4 | Antiviral protein SKI8 OS=Kluyveromyces marxianus (strain DMKU3-1042 / BCC 29191 / NBRC 104275) OX=1003335 GN=SKI8 PE=4 SV=1 |
| W0TEN0 | Zf-C2H2 super family protein OS=Kluyveromyces marxianus (strain DMKU3-1042 / BCC 29191 / NBRC 104275) OX=1003335 GN=AZF1 PE=4 SV=1 |
| W0T601 | U6 snRNA-associated Sm-like protein LSm5 OS=Kluyveromyces marxianus (strain DMKU3-1042 / BCC 29191 / NBRC 104275) OX=1003335 GN=LSM5 PE=3 SV=1 |
| W0T9K4 | Chromatin structure-remodeling complex protein RSC58 OS=Kluyveromyces marxianus (strain DMKU3-1042 / BCC 29191 / NBRC 104275) OX=1003335 GN=RSC58 PE=4 SV=1 |
| W0T789 | NAD-dependent histone deacetylase SIR2 OS=Kluyveromyces marxianus (strain DMKU3-1042 / BCC 29191 / NBRC 104275) OX=1003335 GN=SIR2 PE=3 SV=1 |
