# Supplementary material for: Centromeres in the thermotolerant yeast K. marxianus mediate attachment to a single microtubule
Source: Chromosome Res. 2025 Jul 3;33(1):14. doi: 10.1007/s10577-025-09772-4 (PMC12226651; doi:10.1007/s10577-025-09772-4)
Supplement: Supplementary file 2 — Supplementary file2 (PDF 105 KB) [file 10577_2025_9772_MOESM2_ESM.pdf]

Accession	Protein description
W0T8J4	37S ribosomal protein PET123 OS=Kluyveromyces marxianus (strain DMKU3-1042 / BCC 29191 / NBRC 104275) OX=1003335 GN=PET123 PE=4 SV=1
W0T4D1	LAS seventeen-binding protein 5 OS=Kluyveromyces marxianus (strain DMKU3-1042 / BCC 29191 / NBRC 104275) OX=1003335 GN=LSB5 PE=4 SV=1
W0TGS8	Protein transport protein SEC20 OS=Kluyveromyces marxianus (strain DMKU3-1042 / BCC 29191 / NBRC 104275) OX=1003335 GN=SEC20 PE=3 SV=1
W0TBX6	J protein JJJ2 OS=Kluyveromyces marxianus (strain DMKU3-1042 / BCC 29191 / NBRC 104275) OX=1003335 GN=KLMA_50251 PE=4 SV=1
W0T4J0	Succinate--CoA ligase [ADP-forming] subunit beta, mitochondrial OS=Kluyveromyces marxianus (strain DMKU3-1042 / BCC 29191 / NBRC 104275) OX=1003335 GN=LSC2 PE=3 SV=1
W0TCD3	60S ribosomal export protein NMD3 OS=Kluyveromyces marxianus (strain DMKU3-1042 / BCC 29191 / NBRC 104275) OX=1003335 GN=NMD3 PE=3 SV=1
W0TCI9	Transcription initiation factor TFIID subunit 12 OS=Kluyveromyces marxianus (strain DMKU3-1042 / BCC 29191 / NBRC 104275) OX=1003335 GN=TAF12 PE=3 SV=1
W0T639	37S ribosomal protein S19 OS=Kluyveromyces marxianus (strain DMKU3-1042 / BCC 29191 / NBRC 104275) OX=1003335 GN=RSM19 PE=3 SV=1
W0TDL0	Ribonuclease P protein component OS=Kluyveromyces marxianus (strain DMKU3-1042 / BCC 29191 / NBRC 104275) OX=1003335 GN=RPM2 PE=4 SV=1
W0TGQ9	Nucleoporin NUP120 OS=Kluyveromyces marxianus (strain DMKU3-1042 / BCC 29191 / NBRC 104275) OX=1003335 GN=NUP120 PE=4 SV=1
W0TBS4	Rhomboid protein 1 OS=Kluyveromyces marxianus (strain DMKU3-1042 / BCC 29191 / NBRC 104275) OX=1003335 GN=PCP1 PE=4 SV=1
W0T968	UDP-N-acetylenolpyruvoylglucosamine reductase OS=Kluyveromyces marxianus (strain DMKU3-1042 / BCC 29191 / NBRC 104275) OX=1003335 GN=KLMA_30036 PE=4 SV=1
W0TES5	Transcription elongation factor Spt6 OS=Kluyveromyces marxianus (strain DMKU3-1042 / BCC 29191 / NBRC 104275) OX=1003335 GN=SPT6 PE=3 SV=1
W0TIK1	Target of rapamycin complex 2 subunit TSC11 OS=Kluyveromyces marxianus (strain DMKU3-1042 / BCC 29191 / NBRC 104275) OX=1003335 GN=TSC11 PE=3 SV=1
W0TB40	Origin recognition complex subunit 1 OS=Kluyveromyces marxianus (strain DMKU3-1042 / BCC 29191 / NBRC 104275) OX=1003335 GN=ORC1 PE=3 SV=1
W0TGH0	Ribonuclease P protein subunit RPR2 OS=Kluyveromyces marxianus (strain DMKU3-1042 / BCC 29191 / NBRC 104275) OX=1003335 GN=RPR2 PE=3 SV=2
W0TA59	Methyltransferase domain-containing protein OS=Kluyveromyces marxianus (strain DMKU3-1042 / BCC 29191 / NBRC 104275) OX=1003335 GN=KLMA_40266 PE=4 SV=1
W0TCG9	Superkiller protein 3 OS=Kluyveromyces marxianus (strain DMKU3-1042 / BCC 29191 / NBRC 104275) OX=1003335 GN=SKI3 PE=4 SV=1
W0TED0	CAAX prenyl protease OS=Kluyveromyces marxianus (strain DMKU3-1042 / BCC 29191 / NBRC 104275) OX=1003335 GN=STE24 PE=3 SV=1

W0T6M4	Protein transport protein DSL1 OS=Kluyveromyces marxianus (strain DMKU3-1042 / BCC 29191 / NBRC 104275) OX=1003335 GN=DSL1 PE=4 SV=1
W0TCF5	Target of rapamycin complex 2 subunit BIT61,HbrB super family conserved domain OS=Kluyveromyces marxianus (strain DMKU3-1042 / BCC 29191 / NBRC 104275) OX=1003335 GN=BIT61 PE=4 SV=1
W0TAX8	60S ribosome subunit biogenesis protein NIP7 OS=Kluyveromyces marxianus (strain DMKU3-1042 / BCC 29191 / NBRC 104275) OX=1003335 GN=NIP7 PE=3 SV=1
W0TCJ3	Alanine--tRNA ligase OS=Kluyveromyces marxianus (strain DMKU3-1042 / BCC 29191 / NBRC 104275) OX=1003335 GN=ALA1 PE=3 SV=1
W0T8Z8	Dolichyl-phosphate-mannose--protein mannosyltransferase OS=Kluyveromyces marxianus (strain DMKU3-1042 / BCC 29191 / NBRC 104275) OX=1003335 GN=PMT2 PE=3 SV=1
W0TAY5	Acyltransferase for lyso-phosphatidylethanolamine OS=Kluyveromyces marxianus (strain DMKU3-1042 / BCC 29191 / NBRC 104275) OX=1003335 GN=ALE1 PE=4 SV=1
W0T8C2	RNA polymerase-associated protein LEO1 OS=Kluyveromyces marxianus (strain DMKU3-1042 / BCC 29191 / NBRC 104275) OX=1003335 GN=LEO1 PE=4 SV=1
W0TFE8	Small glutamine-rich tetratricopeptide repeat-containing protein 2 OS=Kluyveromyces marxianus (strain DMKU3-1042 / BCC 29191 / NBRC 104275) OX=1003335 GN=SGT2 PE=3 SV=1
W0THR7	acetyl-CoA C-acetyltransferase OS=Kluyveromyces marxianus (strain DMKU3-1042 / BCC 29191 / NBRC 104275) OX=1003335 GN=ERG10 PE=3 SV=1
W0T4N1	Nuclear pore complex protein Nup85 OS=Kluyveromyces marxianus (strain DMKU3-1042 / BCC 29191 / NBRC 104275) OX=1003335 GN=NUP85 PE=3 SV=1
W0T6Y9	Ribosome assembly protein 3 OS=Kluyveromyces marxianus (strain DMKU3-1042 / BCC 29191 / NBRC 104275) OX=1003335 GN=RSA3 PE=3 SV=1
W0TCE1	Tricalbin-3 OS=Kluyveromyces marxianus (strain DMKU3-1042 / BCC 29191 / NBRC 104275) OX=1003335 GN=TCB3 PE=4 SV=1
W0T8J5	Nucleolar complex-associated protein 3 OS=Kluyveromyces marxianus (strain DMKU3-1042 / BCC 29191 / NBRC 104275) OX=1003335 GN=NOC3 PE=3 SV=1
W0TI30	homocitrate synthase OS=Kluyveromyces marxianus (strain DMKU3-1042 / BCC 29191 / NBRC 104275) OX=1003335 GN=LYS21 PE=3 SV=1
W0T518	Acetyl-CoA hydrolase OS=Kluyveromyces marxianus (strain DMKU3-1042 / BCC 29191 / NBRC 104275) OX=1003335 GN=ACH1 PE=3 SV=1
W0TIH4	CTP synthase OS=Kluyveromyces marxianus (strain DMKU3-1042 / BCC 29191 / NBRC 104275) OX=1003335 GN=URA7 PE=3 SV=1
W0T7P9	Very-long-chain 3-oxoacyl-CoA reductase OS=Kluyveromyces marxianus (strain DMKU3-1042 / BCC 29191 / NBRC 104275) OX=1003335 GN=KLMA_30357 PE=3 SV=1
W0T4S3	U3 small nucleolar RNA-associated protein 11 OS=Kluyveromyces marxianus (strain DMKU3-1042 / BCC 29191 / NBRC 104275) OX=1003335 GN=UTP11 PE=3 SV=1

W0TBC7	phosphoribosylformylglycinamidine synthase OS=Kluyveromyces marxianus (strain DMKU3-1042 / BCC 29191 / NBRC 104275) OX=1003335 GN=ADE6 PE=3 SV=1
W0T9G5	Ribosome production factor 1 OS=Kluyveromyces marxianus (strain DMKU3-1042 / BCC 29191 / NBRC 104275) OX=1003335 GN=RPF1 PE=4 SV=1
